# Atomic Layer Deposition of Buffer Layers for the Growth of Vertically Aligned Carbon Nanotube Arrays

**DOI:** 10.1186/s11671-019-2947-5

**Published:** 2019-04-02

**Authors:** Hao-Hao Li, Guang-Jie Yuan, Bo Shan, Xiao-Xin Zhang, Hong-Ping Ma, Ying-Zhong Tian, Hong-Liang Lu, Johan Liu

**Affiliations:** 1 SMIT Center, School of Automation and Mechanical Engineering, Shanghai University, Shanghai, 201800 People’s Republic of China; 2 State Key Laboratory of ASIC and System, School of Microelectronics, Fudan University, Shanghai, 200433 People’s Republic of China; 3 Shanghai Key Laboratory of Intelligent Manufacturing and Robotics, School of Automation and Mechanical Engineering, Shanghai University, Shanghai, 200072 People’s Republic of China; 4 Electronics Materials and Systems Laboratory, Department of Microtechnology and Nanoscience, Chalmers University of Technology, SE-412 96 Goteborg, Sweden

**Keywords:** Atomic layer deposition, Vertically aligned carbon nanotube arrays, Oxide buffer layers, Thermal interface materials

## Abstract

Vertically aligned carbon nanotube arrays (VACNTs) show a great potential for various applications, such as thermal interface materials (TIMs). Besides the thermally oxidized SiO_2_, atomic layer deposition (ALD) was also used to synthesize oxide buffer layers before the deposition of the catalyst, such as Al_2_O_3_, TiO_2_, and ZnO. The growth of VACNTs was found to be largely dependent on different oxide buffer layers, which generally prevented the diffusion of the catalyst into the substrate. Among them, the thickest and densest VACNTs could be achieved on Al_2_O_3_, and carbon nanotubes were mostly triple-walled. Besides, the deposition temperature was critical to the growth of VACNTs on Al_2_O_3_, and their growth rate obviously reduced above 650 °C, which might be related to the Ostwald ripening of the catalyst nanoparticles or subsurface diffusion of the catalyst. Furthermore, the VACNTs/graphene composite film was prepared as the thermal interface material. The VACNTs and graphene were proved to be the effective vertical and transverse heat transfer pathways in it, respectively.

## Background

Vertically aligned carbon nanotube arrays (VACNTs) have various outstanding performances and show great potential for a wide variety of applications. Due to their high axial thermal conductivity, many VACNT-based thermal interface materials (TIMs) have been developed for thermal packaging applications [1–7]. To synthesize the high-quality VACNTs on different substrates, chemical vapor deposition (CVD) has been commonly used, and the buffer layer should be deposited on the substrate before the deposition of the catalyst, such as Fe. Generally, the buffer layers are used to prevent the diffusion of the catalyst into substrates, so it is also very important to achieve the high-quality buffer layers on different substrates.

Atomic layer deposition (ALD) has self-limited behavior, which could achieve pinhole-free, dense, and conformal films on complex non-planar substrates [8]. Recently, many researchers have used it to deposit the buffer layers for the growth of VACNTs [9–11]. Amama et al. reported the water-assisted CVD of VACNTs using ALD Al as the buffer layer [9]. Quinton et al. reported the floating catalyst CVD of VACNTs using Fe as the catalyst. They found that VACNTs had faster nucleation rate and more uniform tube diameter on ALD Al_2_O_3_ buffer layer, compared with SiO_2_ [10]. Compared with thermal and microwave plasma SiO_2_, the VACNTs grown on ALD SiO_2_ had the fastest nucleation rate [10]. Yang et al. reported that VACNTs could be synthesized on non-planar substrates using ALD Al_2_O_3_ as the buffer layer and Fe_2_O_3_ as the catalyst, respectively [11]. Compared with the planar surface, the non-planar surface could largely increase the specific surface area, which would be very beneficial for the preparation and further applications of VACNTs [12–14]. Although some ALD oxide buffer layers have been synthesized for the growth of VACNTs, their role was still not very clear in the CVD process.

In this research, we used CVD to prepare the VACNTs with different buffer layers, including ALD Al_2_O_3_, ALD TiO_2_, ALD ZnO, and thermally oxidized SiO_2_. The effects of different oxide layers and deposition temperature on the growth of VACNTs were analyzed. Besides, the VACNTs/graphene composite film was also developed as the thermal interface material, and the VACNTs were used as the additional vertical thermal transfer pathways in it.

## Methods

Al_2_O_3_, ZnO, and TiO_2_ thin films were deposited on Si substrates by ALD, and SiO_2_ was formed on Si substrate by thermal oxidization. Trimethylaluminum (TMA), tetrakis(dimethylamino)titanium (TDMAT), and diethylzinc (DEZ) were used as the precursors for ALD of Al_2_O_3_, TiO_2_, and ZnO films, respectively. For all of them, H_2_O was used as the oxygen source, and the deposition temperature was set at 200 °C. The thickness of Al_2_O_3_, ZnO, and TiO_2_, and SiO_2_ films was 20 nm. One-nanometer-thick Fe film was deposited on all of them by electron-beam (EB) evaporation, where it was used as the catalyst. The CVD method was applied to synthesize the VACNTs based on a commercial CVD system (AIXRON Black Magic II). Before the growth of VACNTs, the catalyst was annealed in the hydrogen (H_2_) atmosphere at 600 °C. The period was 3 min, and the flow rate of H_2_ was set at 700 sccm. After that, the acetylene (C_2_H_2_) and H_2_ were introduced into the chamber, and then VACNTs were prepared. The flow rates of C_2_H_2_ and H_2_ were 100 and 700 sccm, respectively. The deposition temperature was changed from 550 to 700 °C, and the period was fixed at 30 min.

**Fig. 1:**
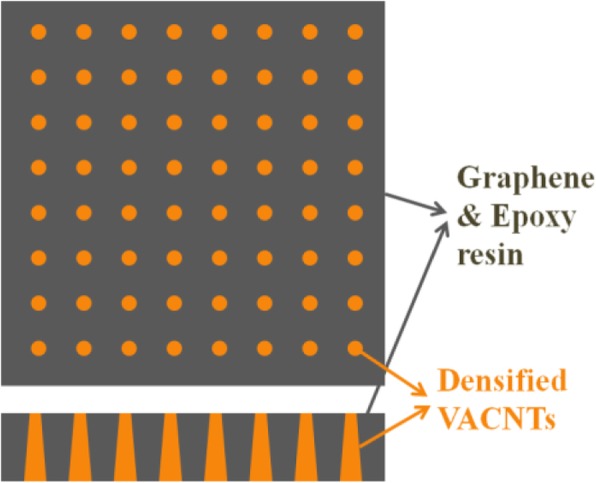
Schematic diagram of the VACNTs/graphene composite film

After the growth of VACNTs on Al_2_O_3_, the VACNTs/graphene composite film was also prepared as the thermal interface material. Epoxy resin, curing agent, and diluents were purchased from Sigma-Aldrich Trading and Tokyo Chemical Industrial Co., Ltd. The multilayer graphene was purchased from Nanjing Xianfeng Nanomaterials Technology Co., Ltd. For the preparation of the composite film, the catalyst was firstly patterned using a lithography machine (URE-2000S/A). The pattern size was 500 μm, and the distance was 150 μm among patterns. Secondly, the VACNTs were deposited by CVD at 650 °C, and the growth period was 30 min. Thirdly, the VACNTs were densified by the acetone vapor, and the period was 20 s. Fourthly, graphene, epoxy resin, curing agent, and diluent were mixed as the matrix, and the amount of graphene was fixed at 10 wt.%. After that, the VACNTs were immersed into the matrix and cured in a vacuum oven at 120 °C for 1 h and then at 150 °C for 1 h. Finally, the prepared composite film was polished to the thickness of about 300 μm, and the tips of VACNTs should be protruded from its both surfaces, as shown in Fig. 1.

The morphology of VACNTs and the composite film was analyzed by the field emission scanning electron microscopy (FESEM, Merlin Compact) and transmission electron microscopy (TEM, Tecnai G2 F20 S-TWIN). Raman spectra of VACNTs were recorded by inVia Reflex, using a laser excitation wavelength of 632.8 nm. The thermal diffusivity (*α*) and specific heat capacity (Cp) of the composite film were measured by the laser flash thermal analyzer (Netzach LFA 467) and differential scanning calorimeter (DSC, Mettler Toledo DSC1), respectively. After that, the thermal conductivity could be calculated according to the Eq. 1:

1 $$ \lambda =\alpha \times \mathrm{Cp}\times \rho, $$

where *λ* and *ρ* were the thermal conductivity and density of the composite film, respectively.

## Results and Discussion

**Fig. 2:**
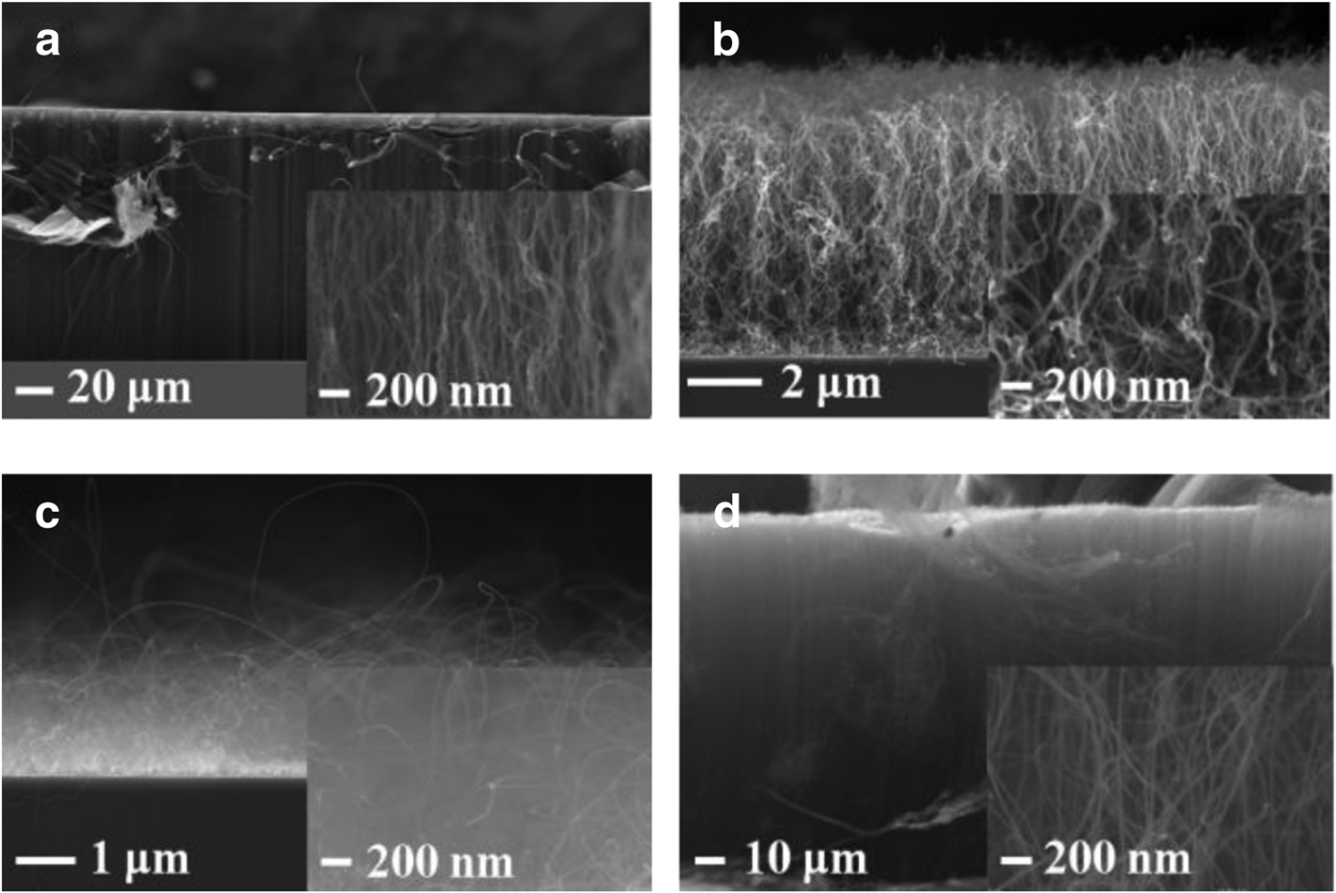
Cross-sectional SEM images of VACNTs grown on different oxide buffer layers at 650 °C: **a** Al_2_O_3_, **b** TiO_2_, **c** ZnO, and **d** SiO_2_

**Fig. 3:**
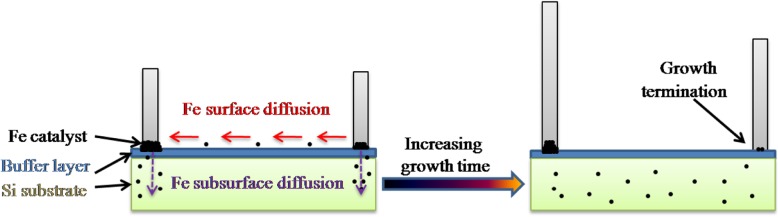
Schematic illustration of Ostwald ripening and subsurface diffusion of Fe catalysts during the growth period of VACNTs

Figure 2 a–d show the cross-sectional SEM images of VACNTs grown on different oxide buffer layers at 650 °C. The VACNTs have been successfully prepared on Al_2_O_3_, TiO_2_, and SiO_2_, as shown in Fig. 2 a, b, and d. Among them, the VACNTs were the thickest on Al_2_O_3_, which indicated that the lifetime of catalyst nanoparticles was the longest on it during the growth period. The lifetime of catalyst nanoparticles represents the time after it has basically lost its catalytic function to grow carbon nanotubes, which could be deduced from the thickness of VACNTs [9]. Unlike it, the relatively thin VACNTs were deposited on SiO_2_ and TiO_2_, which might be caused by the relatively serious Ostwald ripening of catalyst nanoparticles or the subsurface diffusion of Fe [15, 16]. As shown in Fig. 3, Ostwald ripening is a phenomenon whereby larger nanoparticles increase in size while smaller nanoparticles, which have greater strain energy, shrink in size and eventually disappear via atomic surface diffusion [17]. When a catalyst nanoparticle disappeared, or when too much catalyst was lost, the carbon nanotubes growing from it stopped [17]. Besides, subsurface diffusion of Fe into the buffer layer or substrate could also cause mass loss from the catalysts that grow the carbon nanotubes, eventually causing termination of growth [16]. From Fig. 2 a, b, and d, we could also see that the density of VACNTs was the highest on Al_2_O_3_, and the lowest on TiO_2_. Generally, any marginal alignment seen in CVD samples was due to a crowding effect, and carbon nanotubes supported each other by van der Waals attraction [18]. Therefore, it means that the density of VACNTS was quite important, and higher density generally resulted in better vertical alignment of VACNTs, which were confirmed in Fig. 2 a, b, and d. Besides, Fig. 2 c shows that there were almost no VACNTs grown on ZnO, which could be caused by much more serious Ostwald ripening of catalyst nanoparticles and subsurface diffusion of Fe, compared with others [15, 16].

**Fig. 4:**
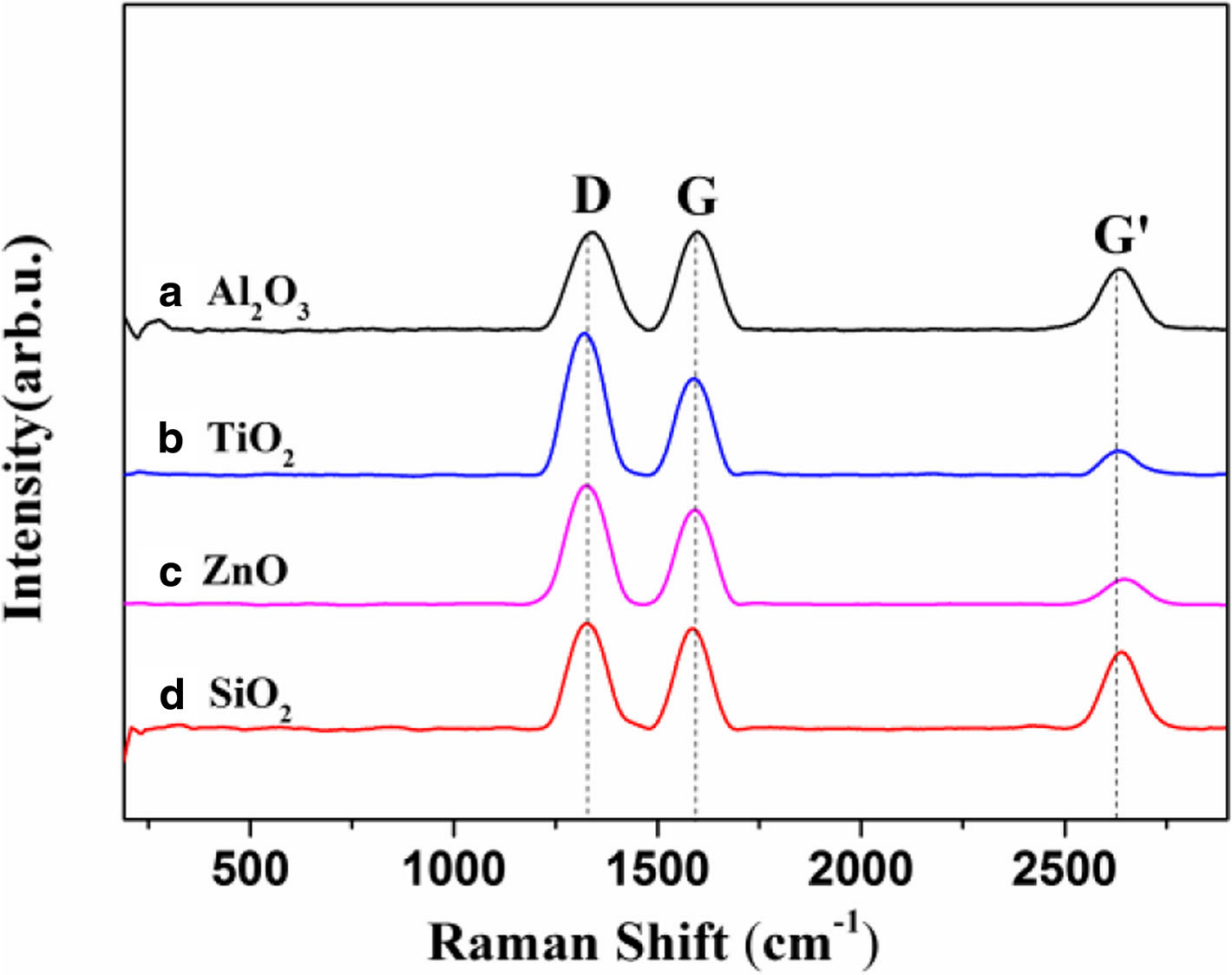
Raman spectra of VACNTs grown on different buffer layers at 650 °C: **a** Al_2_O_3_, **b** TiO_2_, **c** ZnO, and **d** SiO_2_. The spectra have been normalized to the intensity of the G band in order to facilitate comparison

**Fig. 5:**
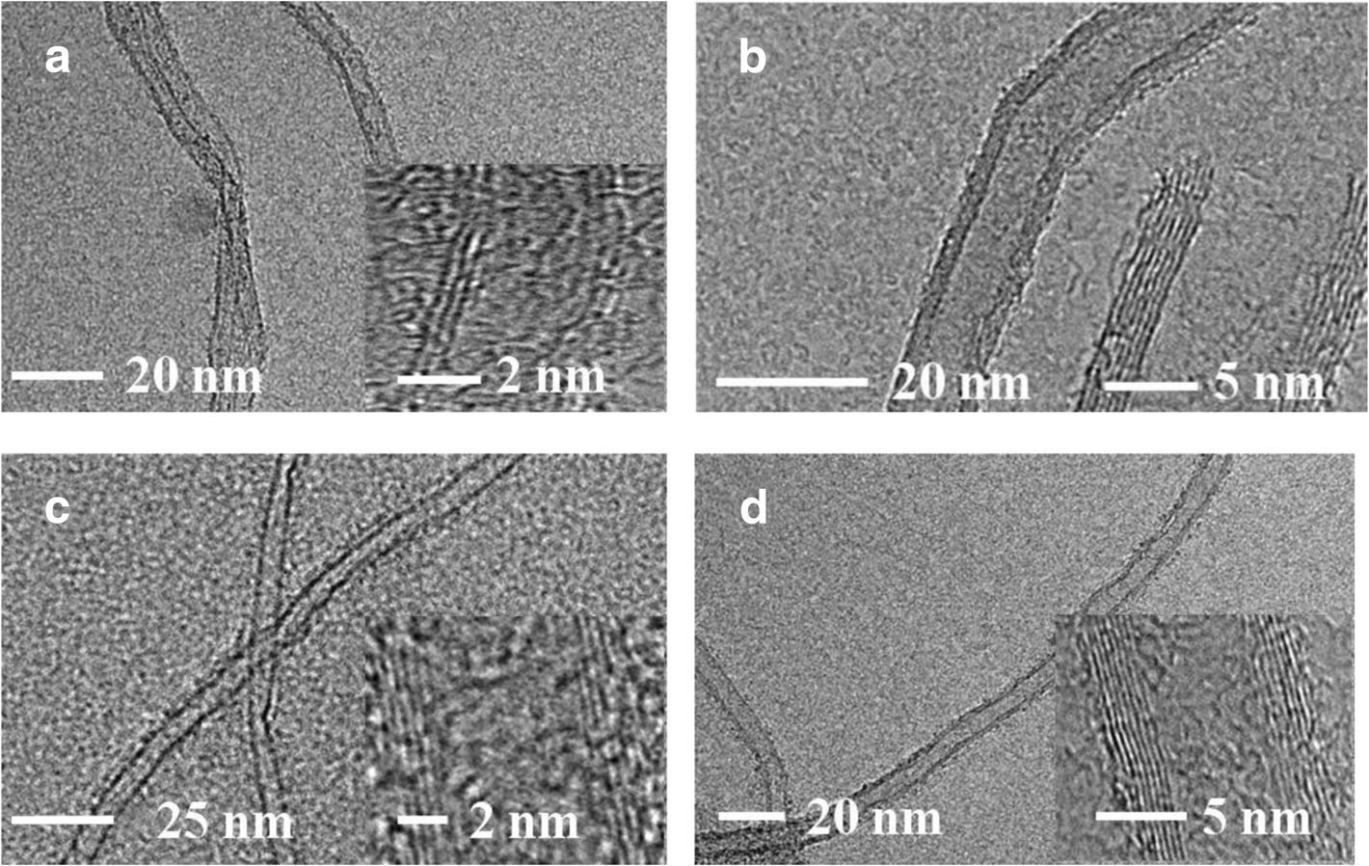
TEM images of VACNTs grown on different buffer layers: **a** Al_2_O_3_, **b** TiO_2_, **c** ZnO, and **d** SiO_2_

Figure 4 a–d show Raman spectra of VACNTs grown on Al_2_O_3_, TiO_2_, ZnO, and SiO_2_. Generally, the D, G, and G’ bands were around 1360 cm^−1^, 1580 cm^−1^, and 2700 cm^−1^, respectively [19, 20]. For different oxide buffer layers, the ratio of *I*_*D*_ and *I*_*G*_ was calculated to be near or more than 1, and there were also no radial breathing modes (RBMs) around 200 cm^−1^. It indicated that all the prepared VACNTs were multi-walled on Al_2_O_3_, TiO_2_, ZnO, and SiO_2_. Figure 5 a–d show the morphology of VACNTs on different oxide buffer layers, which was analyzed by TEM. The VACNTs were multi-walled on all of them, which was consistent with the results of the Raman analysis. The VACNTs were mostly triple-walled on Al_2_O_3_, but more than four walls on TiO_2_, ZnO, and SiO_2_.

**Fig. 6:**
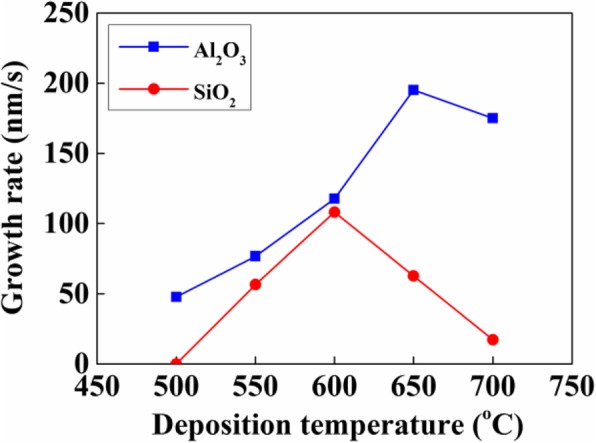
The growth rate of VACNTs variation with deposition temperature on Al_2_O_3_ and SiO_2_ buffer layers

Figure 6 shows the growth rate of VACNT variation with deposition temperature on Al_2_O_3_ and SiO_2_. When the temperature increased, the growth rate of VACNTs firstly raised and then decreased on both of them. It might be related to the serious Ostwald ripening of catalyst nanoparticles or subsurface diffusion of Fe, which largely reduced the lifetime of catalyst nanoparticles and the growth rate of VACNTs [15, 16]. Above 600 °C, the growth rate of VACNTs still increased on Al_2_O_3_, but decreased on SiO_2_. It indicated that the lifetime of catalyst nanoparticles on Al_2_O_3_ was longer than that on SiO_2_. When the deposition temperature was below 500 °C, there were obvious VACNTs on Al_2_O_3_ but no VACNTs on SiO_2_, which meant that the nucleation and initial growth of VACNTs were more easily achieved on Al_2_O_3_, compared with SiO_2_. It indicated that the activation energy for the nucleation and initial growth of VACNTs on Al_2_O_3_ was much lower than that on SiO_2_. Commonly, each catalyst nanoparticle could produce at most one carbon nanotube, but not all the catalyst nanoparticles could achieve the carbon nanotubes, because the activation energy should be overcome for their nucleation and initial growth [21–23]. Therefore, compared with SiO_2_, the lower activation energy of VACNTs on Al_2_O_3_ might result in their higher density, which could be confirmed by Fig. 2 a and d.

**Fig. 7:**
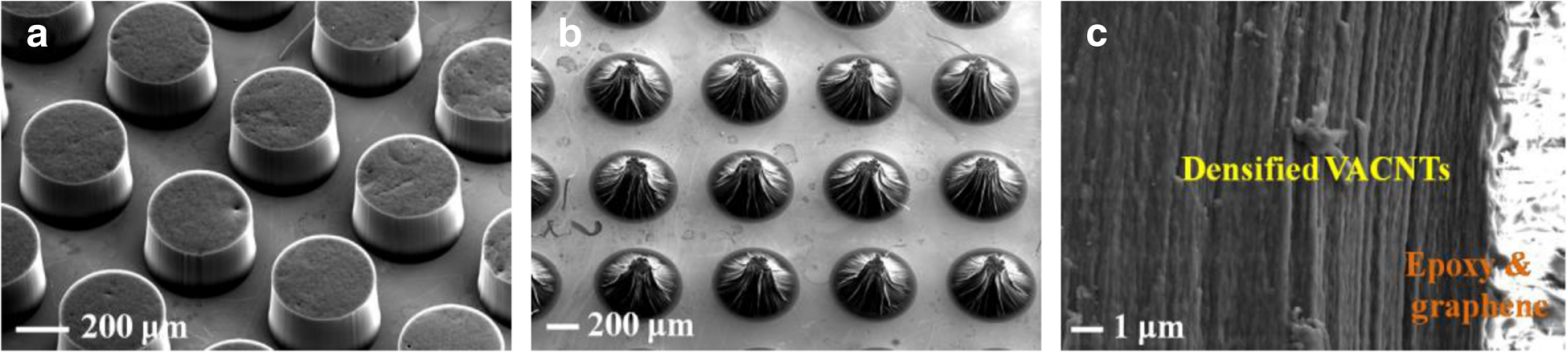
**a** The SEM image of VACNTs with the patterned catalyst. **b** The SEM image of VACNTs after the densification. **c** The cross-sectional SEM image of the VACNTs/graphene composite film

**Fig. 8:**
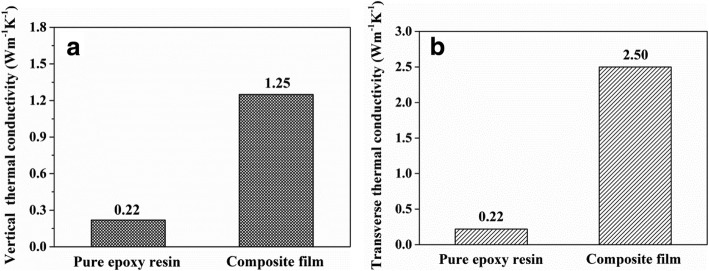
Thermal property of the VACNTs/graphene composite film: **a** the vertical thermal conductivity and **b** the transverse thermal conductivity

Figure 7 a shows the morphology of VACNTs with the patterned catalyst on Al_2_O_3_. Generally, there still had a lot of gaps inside VACNTs, which were filled with air, as shown in Fig. 2 a. However, the thermal conductivity of air was only 0.023 Wm^−1^ K^−1^ at room temperature, so the VACNTs need to be densified to remove it. From Fig. 7 b, we could see that the obvious densification of VACNTs has been achieved with the acetone vapor. Figure 7 c shows the cross-sectional image of the VACNTs/graphene composite film. The VACNTs and graphene were used as the additional vertical and transverse thermal transfer pathways in it. Figure 8 a and b show the vertical and transverse thermal conductivities of the composite film, which were measured to be about 1.25 and 2.50 Wm^−1^ K^−1^, respectively. Compared with the pure epoxy resin, its vertical and transverse thermal conductivities have been obviously enhanced. It confirmed that the effective vertical and transverse heat transfer pathways have been offered by the VACNTs and graphene in the composite film, respectively.

## Conclusions

The growth of VACNTs has been analyzed on different oxide buffer layers, such as ALD Al_2_O_3_, ALD TiO_2_, ALD ZnO, and thermally oxidized SiO_2_. Among them, VACNTs were the thickest and densest on Al_2_O_3_, which indicated that the lifetime of catalyst nanoparticles was the longest and the vertical alignment of VACNTs was the best on it. Besides, the VACNTs were found to be multilayer on Al_2_O_3_, and the deposition temperature was very critical to the growth of VACNTs. Compared with SiO_2_, the nucleation and initial growth of VACNTs were more easily achieved on Al_2_O_3_, which resulted in a higher density of VACNTs on it. After the growth of VACNTs on Al_2_O_3_, they were used to prepare the composite film together with graphene and epoxy resin. Compared with the pure epoxy resin, the vertical and transverse thermal conductivities of the composite film have been largely improved.

## Abbreviations

ALD: Atomic layer deposition
C_2_H_2_: Acetylene
CVD: Chemical vapor deposition
DEZ: Diethylzinc
DSC: Differential scanning calorimeter
EB: Electron-beam
FESEM: Field emission scanning electron microscopy
H_2_: Hydrogen
LFA: Laser flash thermal analyzer
RBMs: Radial breathing modes
TDMAT: Tetrakis(dimethylamino)titanium
TEM: Transmission electron microscopy
TIMs: Thermal interface materials
TMA: Trimethylaluminum
VACNTs: Vertically aligned carbon nanotubes
